# Quality improvement project to reduce medicare 1-day write-offs due to inappropriate admission orders

**DOI:** 10.1186/s12913-024-10594-z

**Published:** 2024-02-14

**Authors:** Olufolarin Oke, K. Michaela Sullivan, Jason Hom, David Svec, Yingjie Weng, Lisa Shieh

**Affiliations:** 1 UT Southwestern Medical Center, Dallas, Texas USA; 2 Stanford Health Care, Palo Alto, California USA; 3 Stanford University School of Medicine, Palo Alto, California USA; 4 Quantitative Sciences Unit, Stanford University School of Medicine, Palo Alto, California USA

**Keywords:** Utilization review, Center for medicare and medicaid services (CMS), Best practice alert (BPA), Medicare 1-day write offs, Inpatient, Outpatient, Observation, CMS 2-midnight benchmark

## Abstract

**Background:**

We identified that Stanford Health Care had a significant number of patients who after discharge are found by the utilization review committee not to meet Center for Mediare and Medicaid Services (CMS) 2-midnight benchmark for inpatient status. Some of the charges incurred during the care of these patients are written-off and known as Medicare 1-day write-offs. This study which aims to evaluate the use of a Best Practice Alert (BPA) feature on the electronic medical record, EPIC, to ensure appropriate designation of a patient’s hospitalization status as either inpatient or outpatient in accordance with Center for Medicare and Medicaid services (CMS) 2 midnight length of stay benchmark thereby reducing the number of associated write-offs.

**Method:**

We incorporated a best practice alert (BPA) into the Epic Electronic Medical Record (EMR) that would prompt the discharging provider and the case manager to review the patients’ inpatient designation prior to discharge and change the patient’s designation to observation when deemed appropriate. Patients who met the inclusion criteria (Patients must have Medicare fee-for-service insurance, inpatient length of stay (LOS) less than 2 midnights, inpatient designation as hospitalization status at time of discharge, was hospitalized to an acute level of care and belonged to one of 37 listed hospital services at the time of signing of the discharge order) were randomized to have the BPA either silent or active over a three-month period from July 18, 2019, to October 18, 2019.

**Result:**

A total of 88 patients were included in this study: 40 in the control arm and 48 in the intervention arm. In the intervention arm, 8 (8/48, 16.7%) had an inpatient status designation despite potentially meeting Medicare guidelines for an observation stay, comparing to 23 patients (23/40, 57.5%) patients in the control group (*p* = 0.001). The estimated number of write-offs in the control arm was 17 (73.9%, out of 23 inpatient patients) while in the intervention arm was 1 (12.5%, out of 8 inpatient patient) after accounting for patients who may have met inpatient criteria for other reasons based on case manager note review.

**Conclusion:**

This is the first time to our knowledge that a BPA has been used in this manner to reduce the number of Medicare 1-day write-offs.

**Supplementary Information:**

The online version contains supplementary material available at 10.1186/s12913-024-10594-z.

## Background

Designation of a patient’s hospital encounter at time of discharge as either outpatient or inpatient, also known as status determination, is important for all parts of the health system including hospitals, insurer, and patients. Outpatient is defined by CMS as a person who has not been admitted as an inpatient but who is registered on the hospital or critical access hospital (CAH) records as an outpatient and receives services (rather than supplies alone) directly from the hospital or CAH [1]. Another designation that is used for patients hospitalized for acute care is called observation. Observation care is care that is provided when additional time for patient testing, monitoring and treatment is needed to help determine if inpatient care is needed and according to CMS, it should be very rare that observation services exceed 48 h and are usually less than 24 h (New referencence) [2]. Observation status is considered outpatient for CMS billing purposes. As a hospital system, status determination has an impact on the amount billed for services provided. This is due to the higher reimbursement rates by the Center for Medicaid and Medicare services (CMS) for encounters designated as inpatient which is billed under Medicare part A and usually reimbursed at a higher rate than services designated as outpatient services which are billed under Medicafare part B [3, 4]. Although services provided to patients hospitalized for inpatient services are almost always of a longer duration than those provided for patients designateted as outpatient, the higher reimbursement rates for inpatient services remains true even when these services are similar to those assigned an outpatient designation. Six of the 10 most common reasons for short inpatient stays were also among the 10 most common reasons for observation stays [3]. However, short inpatient stays were far more costly to Medicare than observation stays [3]. Medicare paid an average of $5,142 per short inpatient stay, but it paid an average of $1,741 per observation stay [3]. To deter hospitals from designating a patient as inpatient when an observation stay may have been appropriate, CMS established the recovery audit program tasked with finding and correcting improper claims to the Medicare program [4]. Data available after creation of this program showed that a large amount of money is being recouped yearly. The amount of money recouped by Medicare based on these programs increased from $939 million in 2011, to $2.4 billion in 2012, to $3.8 billion in 2013 [3]. RAC recoupment reduced in subsequent years for several reasons including hospital’s increased use of appeals and increased compliance but primarily was a result of RAC’s change to the program in 2014 due to industry feedback about the overzealous nature of the RAC program in its previous state [5]. RAC recoupment dropped to $24.33 million in 2017, $73.03 million in 2018 and to $162.03 million in 2019 [6–8].

Inpatient versus outpatient status designation also has financial consequences for the patients too. Patients are often responsible for higher payments under Medicare part B as they may be liable for up to 20% co-insurance for expenses incurred during their stay [9]. Medicare fee-for-service and Medicare advantage enrollees must be provided with the Medicare Outpatient Observation Notice (MOON) according to CMS rules [10]. This notifies patients that they are outpatient receiving observation services and not inpatient.

The classification of a patient as either inpatient or outpatient is made by the patient’s admitting physician but CMS has established a rule to guide physicians known as.

a length of stay benchmark [2, 11]. Providers billing Medicare for services are encouraged to follow this benchmark in determining a patient’s status at time of hospitalization as either inpatient or Observation. This information can be found on the Inpatient Prospective Payment System (IPPS-2014) final rule and states that a provider should designate patients whose hospitalization are expected to span less than two midnights as outpatient based on medical necessity with two notable exemptions: Procedures appearing on the CMS’s inpatient-only procedure list and a “rare and unusual” circumstance in which inpatient admission would be reasonable regardless of length of stay [12]. Due to the complexity of status determinations and the monetary advantage an inpatient designation confers, it is not surprising to see large variability between hospitals in the application of outpatient versus inpatient status [3, 6]. To assist physicians with making this occasionally complex decision and as required by Medicare Conditions of Participation, most hospital systems establish a utilization review (UR) team which often comprises of physicians, nurse case managers and physician advisors who have higher levels of expertise with the insurance related rules. Commercial tools such as InterQual (McKesson Corporation, San Francisco, CA) and MCG (formally known as Milliman Care Guidelines; MCG Health, LLC, Seattle, WA) are also available to help define inpatients versus outpatients [13, 14] but CMS guidelines including the 2-midnight LOS benchmark takes precedence over these tools.

At Stanford Health Care, ensuring compliance with CMS guidelines on hospitalization status (Inpatient versus outpatient) is monitored by the UR team which comprises of nurse case managers, providers and physician advisors with expertise in insurance and CMS guidelines. Nurse case Managers review each patient’s designation within twenty-four hours of hospitalization and prior to discharge for compliance with CMS criteria for an inpatient stay with a physician advisor readily available for more complex cases. If a patient is admitted as inpatient and does not meet length of stay criteria or any of the exceptions listed on Medicare guidelines, the UR team including the nurse case manager can then make recommendations to the patient’s treating provider who ultimately is responsible for making the decision on whether the patient should remain in inpatient admission status or if the patient should be changed to an Observation status.” The billing code associated with this change in status is known by CMS as a “condition code 44” [1]. After a condition code 44 is performed, SHC bills for outpatient services and is reimbursed based on Medicare fee service for outpatient services (Medicare Outpatient Prospective Payment Schedule). Unless the patient requires observation services for at least 8 h, the inpatient part B charges are not captured or billed.

Despite the efforts of our nurse case managers and UR team, there are often cases of patients who are admitted and discharged in an inpatient admission status but upon retrospective review prior to billing CMS are found not to meet the medical necessity or the length of stay benchmark for an inpatient stay. CMS can deny these claims if billed under Medicare Part A for inpatient services if they also find them not reasonable and necessary [11].

In accordance with CMS rules, SHC does not bill Medicare under Part A for these patients. Instead, Stanford, under a process known as self-denial, bills for part B services for some ancillary services that qualify per Medicare rules called “Type of bill-121” [15, 16]. The remaining charges for services provided but not billed to Medicare are considered a write-off.

These Medicare 1-day write-offs lead to significant annual income loss for Stanford Health Care. Charge data for write-offs for fiscal year 2016–2018 were $10.8 M, $3.4 M, and $2.6 M. It should be noted that Charge data is often significantly higher than Medicare allowable charges/re-imbursement.

To reduce these Medicare 1-day write-offs, we sought to take advantage of the electronic health record system, in our case (EPIC), to incorporate a tool called best practice alert (BPA) that has been shown to be effective in the past by other healthcare systems [17–19] in both inpatient and outpatient setting for a variety of interventions. BPA has been used successfully to Improve efficient utilization use of resources within healthcare system, change patient clinical outcomes for specific conditions such as sepsis and increase appropriate medication use in primary care clinic setting for common medical co-morbidities such as hypertension and diabetes.

Researchers at University of Florida conducted a three-year study from 2014 to 2017 that examined the efficacy of EMR BPA in reducing repetitive laboratory test and hospital cost [11]. The intervention reduced the overall duplicates by 18% (OR = 0.82, standard error + 0.16, P-value < 0.000). In addition, in this study, the estimated cost savings was about $72,543 over 17 months in the post-intervention period [17].

A prospective study done in a University of Virginia surgical/trauma/burn ICU using a BPA aimed at identifying with patients with septic shock and promoting timely administration of antibiotics [19]. The study showed that there was a trend towards decreased time-to-antibiotics following implementation of the BPA (7.4vs4.2 h, *p* = 0.057) [18].

Another study done at eight UCLA primary care setting in 2013 aimed to assess provider responses to a focused BPA alert for the intensification of blood pressure medications before versus after implementation of the chart closure hard stop (20). Results showed that among BPA that represent clear opportunities for treatment, providers ordered the indicated medication more often (41% vs. 75%) after the “chart closure” hard stop was implemented (*P* = 0.01) [19].

Based on the success of these studies, we aimed to incorporate a BPA tool into Epic with the goal of improving compliance with Medicare rules regarding appropriate inpatient status designation to Medicare 1 day write-off at Stanford Health Care.

## Methods

### Setting

The randomized control phase of the study took place at Stanford Health Care (SHC) which comprises of SHC-Palo Alto located in Stanford, California, and SHC-Valley Care (SHC-VA) located in Pleasanton, California, between July 18, 2019, and October 18, 2019. The BPA was non-randomized to all non-surgical services after the BPA was completed. Independent of this study, SHC collects data regarding 1-day Medicare write-offs and reported Medicare 1-day write-offs in charge dollars for every fiscal year from 2019 to 2023 as part of our BPA effect confirmation.

Patients included in the study must meet all the inclusion criteria which were: Patients must have Medicare fee for service insurance, inpatient length of stay (LOS) less than 2 midnights (Zero to one day), inpatient status designation at time of discharge, was admitted to an acute level of care and belonged to one of 37 listed hospital services at the time of signing of the discharge order. Inpatient length of stay spans the time from admission order placement by ED provider to discharge order placement. The names of the 37 non-surgical services are listed in Appendix 1. An IRB waiver (Protocol number 70,191) was obtained on a retroactive basis on 4/26/23 based on the classification of this study as quality improvement.

### Problem definition

**Fig. 1:**
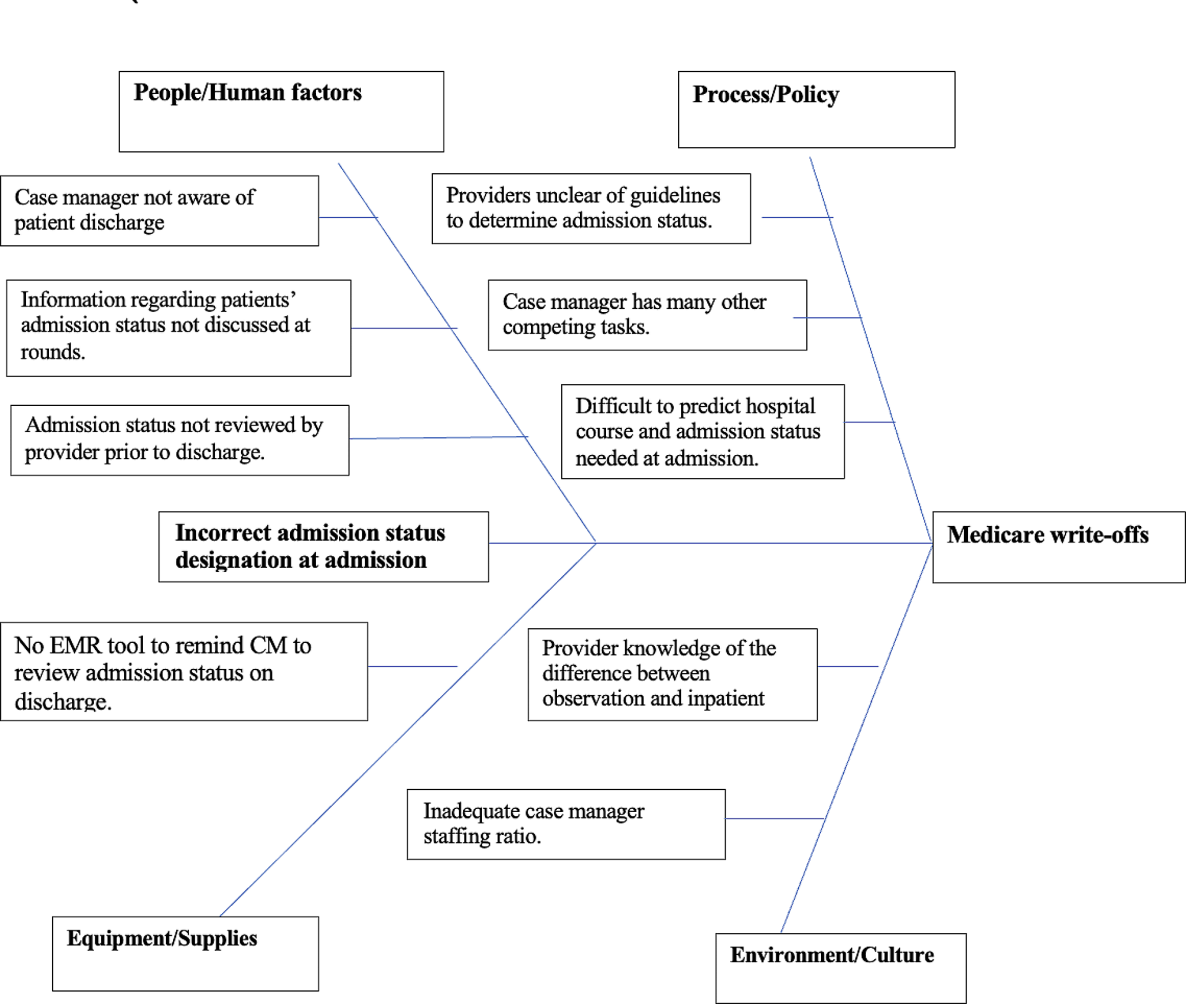
Fishbone Analysis

**Fig. 2:**
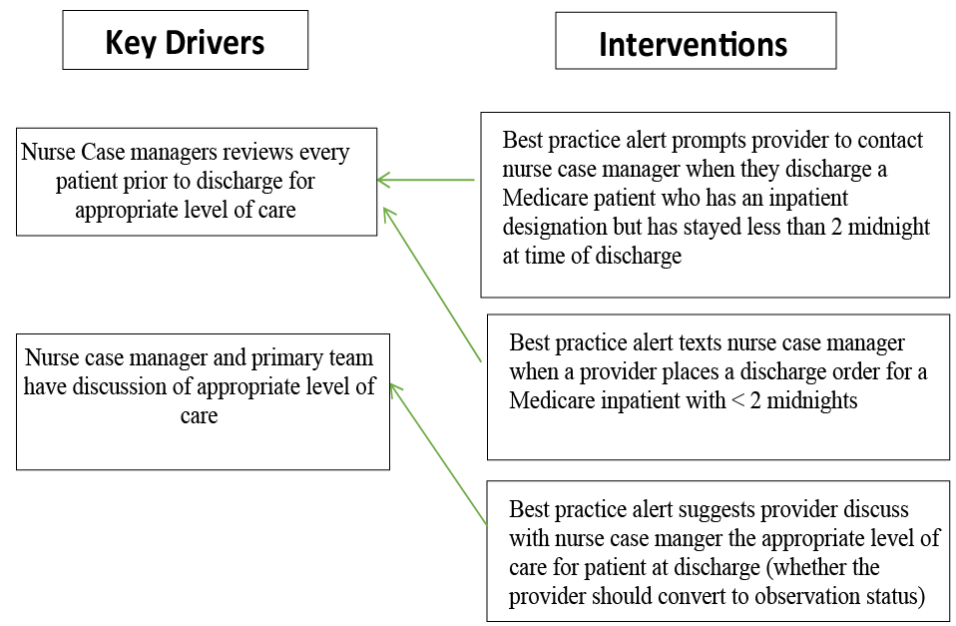
Key drivers and interventions

We identified that Stanford Health Care had a significant number of patients who after discharge are found by the UR committee not to meet CMS 2-midnight benchmark for inpatient status. Some of the charges incurred during the care of these patients are written-off and known as Medicare 1-day write-offs. We began by performing a root cause analysis using A3 methodology and a fish bone analysis as seen in Fig. 1 to identify what intervention would best address the issue of income loss due to Medicare 1- day write-offs. The primary initial event was incorrect designation of a patient at time of hospitalization as either observation or inpatient. The two key drivers identified in this process were the primary provider team and the nurse case manager responsible for the patient as shown in Fig. 2. If neither the primary team nor the nurse case manager intervened in changing the patients’ designations prior to discharge, this would likely result in a Medicare write-off.

We identified several human factors, process/policy, equipment/supplies, and environmental/cultural issues in the Stanford healthcare system that contributed to the failure to designate a patient the appropriate hospitalization status. After evaluating each one of these factors, the intervention we identified that is most likely to achieve our goal of improving compliance with Medicare 2 midnight rule regarding the appropriate designation of patients in an inpatient status thus reducing Medicare write-offs in the Stanford health care system in the inpatient setting was the introduction of a best practice alert (BPA) in the EPIC electronic medical record system. The EPIC BPA would alert providers to resolve any discrepancy between a patient’s length of stay, the complexity of primary hospital problem and the designated hospitalization status during the discharge process but prior to completing discharge in compliance with Medicare guidelines.

### Intervention creation and implementation

A best practice advisory (BPA) intervention was co-developed the help of our EPIC EMR and utilization review team including a member who was an expert in Medicare compliance. Pre-specified inclusion criteria are: Patients must have Medicare fee for service insurance, inpatient length of stay (LOS) less than 2 midnights, inpatient status designation at time of discharge, hospitalization to an acute level of care and belonged to one of 37 listed hospital services listed in Appendix 1 at the time of signing of the discharge order.

The BPA which can be seen in Figure S1 fires when a provider places a discharge order for a patient who meets the inclusion criteria as stated above. The BPA would prompt the provider to discuss the patient’s inpatient status with the utilization review nurse case manager who also receives an alert. The utilization review nurse case manager consults with the utilization management team who then reviews the patient’s hospital stay for medical necessity to ensure they meet CMS’s established criteria for inpatient status designation. The utilization review’s recommendation is then passed on to the provider who attempted to place the discharge order. If the recommendation of the UR team is a change in status from inpatient to observation and the primary team agrees, the discharge order is cancelled, and the condition Code 44 process would occur. The previous inpatient order is cancelled, and an observation order would be placed followed by a new discharge order. Given that the total hospitalization time in observation status is essentnially zero, SHC does not bill CMS for comprehensive observation services.

Of note, the primary team has the option to bypass the BPA and the reason for BPA override reason would be noted. All patients that were admitted between July 18, 2019, and October 18, 2019, to SHC-Palo Alto and SHC Valley Care Medical Center were randomized to either the intervention arm (BPA fires) or control (BPA silent). A total of 88 patients met this inclusion criteria.

Chart review was then conducted and information regarding each patient’s designation as either inpatient or outpatient at time of discharge was documented. Nurse case manager notes were also reviewed for each patient to obtain information regarding whether inpatient designation was appropriate based on other criteria that may not have been known by the discharging provider. The BPA was then activated in EPIC for all patients in fiscal year 2019 and income loss attributable to Medicare 1-day write-off was obtained.

The primary outcome was the estimated number of write-offs in both intervention and control arm while the secondary outcome was the number of patients assigned observation status at time of discharge compared to those assigned an inpatient status.

### Statistical analysis

We reported the frequencies and proportions of patients who were ultimately assigned to inpatient vs. observation status at discharge by the intervention (Epic BPA) and control (non-Epic BPA) groups respectively. The difference of the proportions of patients assigned to observation status was compared by the intervention group to the control group and reported along with the 95% confidence interval. We further reported frequencies and proportions write-off in patients who were assigned to the inpatient status by the intervention and control group. Fisher’s exact tests were performed to evaluate the differences between two groups in both discharge status assignment and write-offs, using a free online tool which can be found here (https://www.socscistatistics.com/tests/fisher/default2.aspx). *p* < 0.05 is considered as statistically significant.

BPA Efficacy was calculated as the percentage of patients in the intervention group who were appropriately designated correctly either as inpatient or observation at the end of the study.

### Effect confirmation

Review Medicare write-offs at the end of the fiscal year 2019 and 2020 to determine if the BPA has the effect that would be expected based on the study results above.

### Sustain plan

Utilization management team’s monthly review of BPA data to ensure that providers are being reminded by nurse case managers to change patient’s designation from inpatient to observation when deemed appropriate.

## Results

**Fig. 3:**
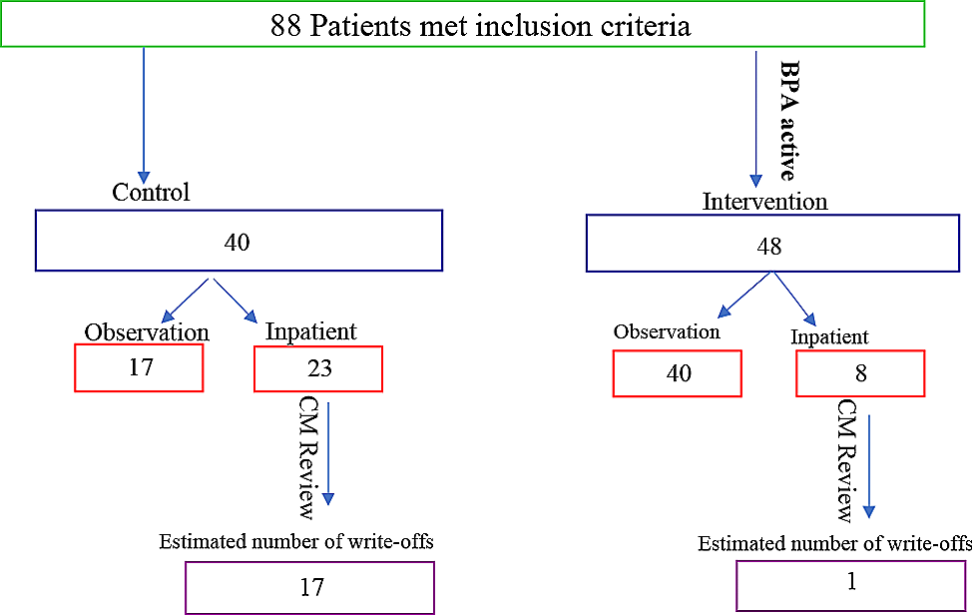
Flow chart showing estimated number of write offs in the intervention and control arms of the study

A total of 88 patients were included in this study: 40 in the control arm and 48 in the intervention arm. In the intervention arm, 8 (8/48, 16.7%) had an inpatient status designation despite potentially meeting Medicare guidelines for an observation stay, comparing to 23 patients (23/40, 57.5%) patients in the control group, which is statistically significant (*p* = 0.001) (Fig. 3; Table 1).

**Table 1:** The number of patients with observation status versus inpatient status in intervention and interventional arm

	Intervention (*n* = 48)	Control (*n* = 40)	Difference in % Observation Patient (Intervention-Control) [95% Confidence Interval]	P value*
Inpatient	8 (16.7%)	23 (57.5%)	40.8% [22.4%, 59.4%]	0.0001
Observation	40 (83.3%)	17 (42.5%)		

*P value based on fisher’s exact test

The estimated number of write-offs in the control arm was 17 (73.9%, out of 23 inpatient patients) while in the intervention arm was 1 (12.5%, out of 8 inpatient patient) after accounting for patients who may have met inpatient criteria for other reasons based on nurse case manager note review (Fig. 3).

Estimated number of write-offs was calculated using the number of patients that were not converted from inpatient to observation as a surrogate after deducting the number of patients who the nurse case manager stated met inpatient criteria based on chart review.

The percentage of patients who met inpatient criteria in the intervention arm based on chart review was used to calculate the number of patients who would have met such criteria in the control arm if all patients had been reviewed by the nurse case manager. This is because the active BPA in the intervention arm ensured that nurse case managers reviewed the patients prior to discharge. The percentage of patients who met inpatient criteria in the intervention arm (7/48 = 14.58%). Therefore, about 6 patients (0.1458*40) was calculated to have met inpatient criteria in the observation group leading to 17 (23 − 6) estimated number of write-offs in the control group.

The estimated value of the 1-day Medicare write-off averted because of the BPA is estimated to be about $329,088($5,142*16*4). This calculation is based on historical data from CMS who paid an average of $5,142 for short inpatient stays.

BPA efficacy in the intervention group was 98% (47/48). On chart review, provider for the one patient not converted to observation bypassed the BPA with comment “Will discuss with CM” but no nurse case manager note was seen so it was unclear the reason why patient was left in an inpatient status.

Data from SHC in the subsequent years showed that Medicare 1-day stay write-off charge dollars were much lower compared to the years before the BPA. The write-off charge data were $1.0 M for FY 2019, 1.07 M for FY 2020, $792K for FY 2021, $551K for FY 2022 and $571K for FY23 comapred to $10.8 M, $3.4 M, and $2.6 M for fiscal years 2016, 2017 and 2018 respeectively. Of note, charge data is often much higher than allowable Medicare charges which would represent actual Medicare 1-day write-off amount in dollars.

## Discussion

We incorporated a BPA to supplement the efforts of patient’s treatment provider, nurse case managers and UR team in ensuring compliance with CMS 2 midnight rule benchmark for designation of patients as inpatient or observation (Outpatient) status thereby reducing 1-day Medicare write-offs.

Like other prior studies that have shown the effectiveness of BPA as a tool with diverse impact on improving several aspects of the functioning of a healthcare system [17–19], our study showed that a BPA can be effective as a supplementary tool to improve compliance with guidelines in our case Medicare 2 midnight benchmark for appropriate status designation. In addition, the effects of the BPA have been sustained for several years since completion of the study in 2019 as Medicare 1-day write-offs post intervention (FY 2019 onwards) has remained consistently lower than years before the BPA was instituted.

In addition, this BPA also provides an opportunity to improve patient satisfaction in our health care system. Given that patients billed under Medicare part B often have a higher out of pocket medical bill than those billed under Medicare part A [7], using the condition code 44 process prior to discharge and providing patients with the “MOON” prepares patients as opposed to a surprise bill they may receive in the mail if a Part A self-denial is done by the hospital with retrospective billing for Medicare part B services.

Condition code 44 billing process provides additional benefit such as the concurrent review process falls within the natural workflow of the case management and UR team workflow process as opposed to a retrospective review. One of the most important aspects of utilizing a condition code 44 is to increase compliance with CMS guidelines which can lessen the risk of CMS audit for inpatient services given that condition code 44 is billed as outpatient as opposed to inpatient. Another advantage that is gained is that there is a shorter time to reimbursement when a condition code 44 process is utilized allowing hospitals to be able to allocate resources and complete other necessary projects more efficiently. Lastly, there is no effect on re-admission data for patients appropriately placed in observation status.

The BPA can be potentially modified and expanded to other status determination scenarios such as the capturing of patients in observation status who may qualify for inpatient status and medically complex patients designated as hospitalized surgical outpatient who spend at least one night in the hospital for routine recovery who may qualify for inpatient. At SHC, these cases would be referred to our physician advisor and UR team for further review.

The success of the BPA and its continued effectiveness relies greatly on full participation from all the members involved in the process which included admitting providers, nurse case managers, physician advisors and other members of the UR team. Other healthcare systems who wish to incorporate similar BPA should ensure that all the team members are fully invested and willing to participate in the intervention otherwise the efficacy rate of the intervention will likely decrease. In the future, there is potential for other types of BPA to be used in addressing other issues faced within SHC.

### Limitations

One main limitation of the study is that the estimated 1-day Medicare write-off is an over-estimation. This is because charge data as reported in this study is often much higher than Medicare allowable charges which would represent the true write-off dollars. However, our finance department does apply a formula based on expected payment from that financial class to “value” the adjustment. Nonetheless, the improved compliance with Medicare 2 midnight benchmark guidelines is our desired outcome and remains a benefit.

BPA efficacy rate was less than 100% (98%) as the BPA is not a hard stop. Providers under the pressure of time may choose to bypass the Epic BPA and if case managers are not available nor assigned to the team, they may not get the alert or be able to get in touch with providers in time prior to patient discharge. This can be alleviated by also alerting a dedicated utilization management team member simultaneously to follow up in real time if the case manager is unable to do so but this may strain resources and increase workload.

## Conclusion

We are the first to incorporate this Epic BPA tool into the discharge workflow and show its positive sustained effect in reducing Medicare 1-day write-offs. We have expanded the alerting process to a dedicated UR member for all services and health insurances.

### Electronic supplementary material

Below is the link to the electronic supplementary material.

**Supplementary Material 1: Figure S1:** Screen shot of Epic BPA

**Supplementary Material 2: Appendix 1:** List of 37 non-surgical hospital services included in the study

## Data Availability

The datasets used and/or analyzed during the current study are available from the corresponding author on reasonable request.

## Abbreviations

BPA: Best Practice Alert
SHC: Stanford Health Care
CMS: Center for Medicaid and Medicare Services
IPPS: Inpatient Prospective Payment System
EMR: Electronic Medical Record
RAC: Recovery Audit Program
