# Supplementary figures and images for: Quality improvement project to reduce medicare 1-day write-offs due to inappropriate admission orders
Source: BMC Health Serv Res. 2024 Feb 14;24:204. doi: 10.1186/s12913-024-10594-z (PMC10868014; doi:10.1186/s12913-024-10594-z)

**Figure S1: Screen shot of Epic BPA**


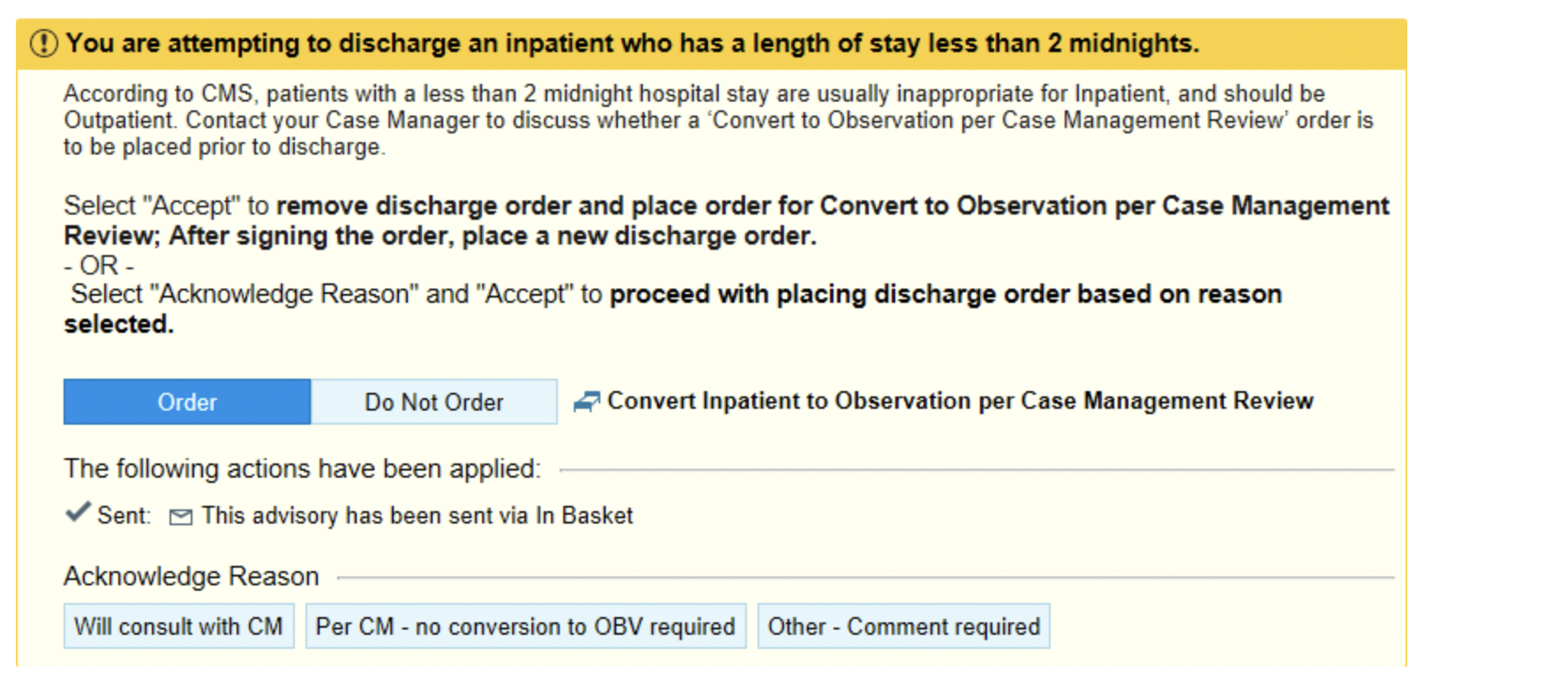


© 2022 Epic Systems Corporation

Supplement: Supplementary file 1 — Supplementary Material 1: Figure S1: Screen shot of Epic BPA [file 12913_2024_10594_MOESM1_ESM.docx]

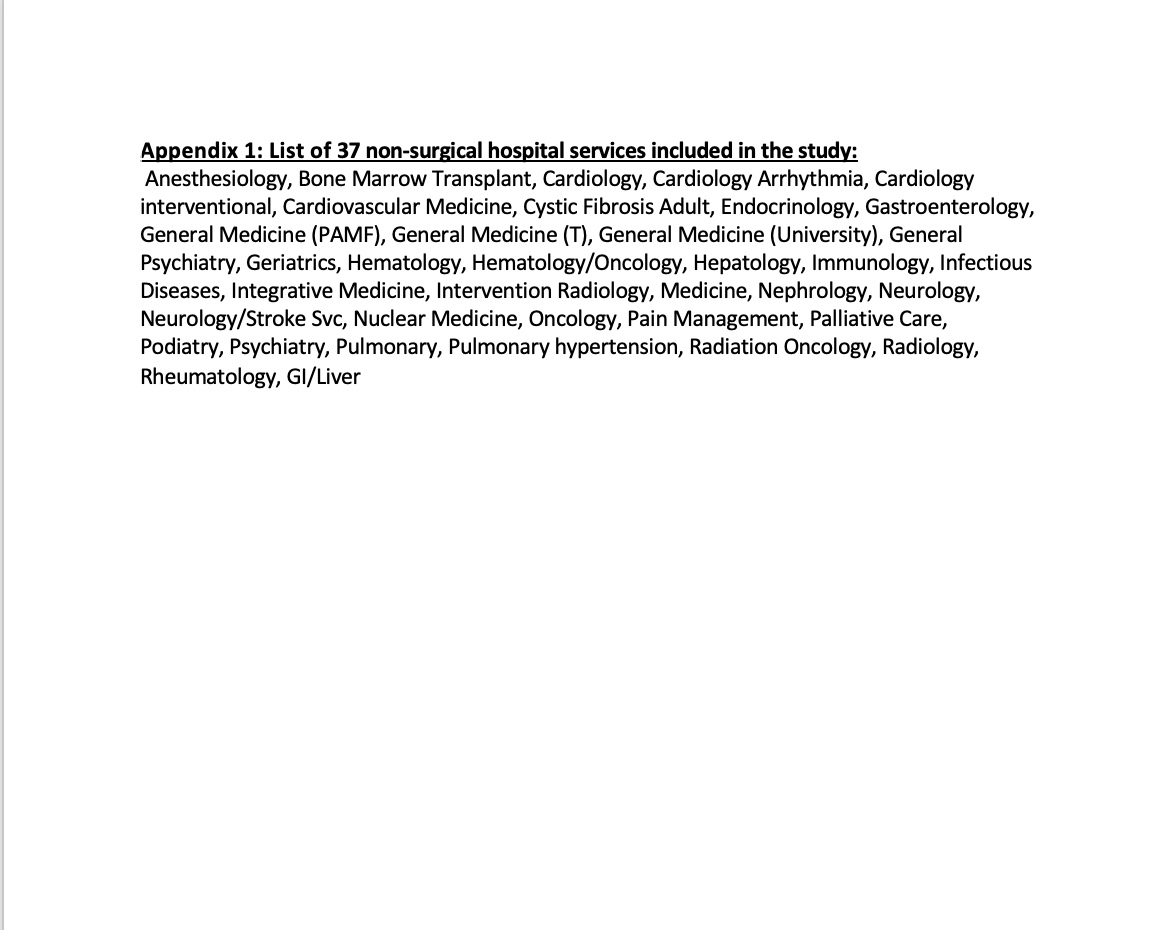

Supplement: Supplementary file 2 — Supplementary Material 2: Appendix 1: List of 37 non-surgical hospital services included in the study [file 12913_2024_10594_MOESM2_ESM.jpg]
